# Differential contribution of TrkB and p75^NTR^ to BDNF-dependent self-renewal, proliferation, and differentiation of adult neural stem cells

**DOI:** 10.3389/fnmol.2023.1271820

**Published:** 2023-12-22

**Authors:** Anna Lozano-Ureña, José M. Frade

**Affiliations:** Laboratory of Neuronal Generation and Degeneration in Vertebrates, Department of Molecular, Cellular and Developmental Neurobiology, Cajal Institute, CSIC, Madrid, Spain

**Keywords:** SVZ, self-renewal, neurosphere, neurotrophin, TrkB.T1, oligodendrocyte, neuron, co-receptor dependence

## Abstract

Alterations in adult neurogenesis are a common hallmark of neurodegenerative diseases. Therefore, understanding the molecular mechanisms that control this process is an indispensable requirement for designing therapeutic interventions addressing neurodegeneration. Neurotrophins have been implicated in multiple functions including proliferation, survival, and differentiation of the neural stem cells (NSCs), thereby being good candidates for therapeutic intervention. Brain-derived neurotrophic factor (BDNF) belongs to the neurotrophin family and has been proven to promote neurogenesis in the subgranular zone. However, the effects of BDNF in the adult subventricular zone (SVZ) still remain unclear due to contradictory results. Using *in vitro* cultures of adult NSCs isolated from the mouse SVZ, we show that low concentrations of BDNF are able to promote self-renewal and proliferation in these cells by activating the tropomyosin-related kinase B (TrkB) receptor. However, higher concentrations of BDNF that can bind the p75 neurotrophin receptor (p75^NTR^) potentiate TrkB-dependent self-renewal and proliferation and promote differentiation of the adult NSCs, suggesting different molecular mechanisms in BDNF-promoting proliferation and differentiation. The use of an antagonist for p75^NTR^ reduces the increment in NSC proliferation and commitment to the oligodendrocyte lineage. Our data support a fundamental role for both receptors, TrkB and p75^NTR^, in the regulation of NSC behavior.

## 1 Introduction

Two main regions maintain the potential to generate new neurons in the adult mammalian brain: the subventricular zone (SVZ) in the wall of the lateral ventricles and the subgranular zone (SGZ) in the dentate gyrus of the hippocampus (Taupin and Gage, 2002; Chaker et al., 2016; Gonçalves et al., 2016). The neural stem cells (NSCs) are not only responsible for carrying out this process of neurogenesis but also contribute to generating new astrocytes and oligodendrocytes throughout life (Taupin and Gage, 2002; Sohn et al., 2015), thus becoming potential agents for brain repair. Under homeostatic conditions, a careful interchange between cellular and molecular processes in the microenvironment constantly regulates the activity of NSCs (Fuentealba et al., 2012). In order to avoid loss or excess of the stem cell (SC) population, self-renewal and proliferation must be acutely regulated in coordination with differentiation processes. Thus, the differentiation of NSCs requires an intermediate state in which these cells become committed [i.e., neural progenitor cells (NPCs)] although they still show proliferative potential (Llorente et al., 2022).

Brain-derived neurotrophic factor (BDNF) is the most widely distributed member of the neurotrophin (NT) family in the central nervous system (Leibrock et al., 1989), with important implications in neuronal survival and differentiation (Eide et al., 1993). BDNF interacts with two receptors, the tropomyosin-related kinase B (TrkB) receptor (Klein et al., 1989) and the p75 neurotrophin receptor (p75^NTR^), known to interact with all NTs (Rodriguez-Tebar et al., 1992). Classically, NTs promote survival, proliferation, and correct maturation by Trk receptor signaling through its associated kinase activity (Mitra et al., 1987), whereas p75^NTR^ has been more involved in apoptosis (Frade et al., 1996) and in other cellular pathways depending on the intracellular complexes it constitutes (Roux and Barker, 2002). A recent study has begun to clarify the complexity of p75^NTR^ signaling. This includes proteolytic processing through γ-secretase to release its intracellular domain (Vicario et al., 2015) that translocates to the nucleus (Parkhurst et al., 2010) and the conformational rearrangement of disulfide-linked receptor dimers (Klein et al., 1990) that allows the access of intracellular effectors to the receptor (Lin et al., 2015). BDNF, TrkB, and its truncated form TrkB.T1, known to lack the kinase domain (Klein et al., 1990), are all expressed in the murine SVZ (Vilar and Mira, 2016) as well as throughout the migratory pathway (Chiaramello et al., 2007). p75^NTR^ is also expressed by cycling cells of the SVZ (Okano et al., 1996; Giuliani et al., 2004), including intermediate progenitors (Galvão et al., 2008). In addition, p75^NTR^ can be detected in neuroblasts of the SVZ/RMS (Galvão et al., 2008), and genetic depletion of p75^NTR^ reduces the migration capacity of the neuroprogenitors in the SVZ both in physiological conditions and after cortical injury (Young et al., 2007; Deshpande et al., 2022). The complexity of NT signaling is increased due to the known association of p75^NTR^ with members of the Trk family (Hempstead et al., 1991; Zanin et al., 2019). This is also the case for BDNF as the treatment with BDNF in embryonic hippocampal neurons elicits the association of TrkB and p75^NTR^, facilitating the TrkB signaling and promoting neuronal survival and function (Zanin et al., 2019).

The activity of BDNF by the high-affinity binding to TrkB has been widely described in the hippocampal neurogenic niche (Bartkowska et al., 2007; Li et al., 2008; Vilar and Mira, 2016); however, its role in the NSCs located at the SVZ is not fully understood (Bath et al., 2012; Vilar and Mira, 2016). Although both BDNF receptors, TrkB and p75^NTR^, are present in the adult SVZ (Tervonen et al., 2006; Galvão et al., 2008; Bath et al., 2012; Vilar and Mira, 2016), the implication of these receptors in NSC decision-making remains to be established. BDNF/TrkB participates in the proliferation and differentiation of the neuroprogenitors, and in the survival and maturation of the new neurons (Berghuis et al., 2006; Bath et al., 2012; Chen et al., 2013). BDNF/p75^NTR^ seems to regulate cell proliferation and migration of neuroblasts to the olfactory bulb (OB) (Snapyan et al., 2009; Bath et al., 2012; Deshpande et al., 2022).

Alterations in the niche environment as a consequence of stroke or neurodegenerative diseases, among others, drive a disorder in the amount of BDNF and its receptors (Holsinger et al., 2000; Jiao et al., 2016; Deshpande et al., 2022). These changes in BDNF concentration might imply the activation of different signaling pathways and, thus, the different context-dependent effects observed in previous studies (Bath et al., 2012). Investigating the function of BDNF and the molecular mechanisms implicated in the regulation of adult NSCs is essential to understand the potential contribution of adult NSCs to brain repair and as a therapeutic tool. Here, we analyzed the effect of low and high concentrations of BDNF in the self-renewal, proliferation, and differentiation capacity of NSCs isolated from the adult SVZ and the contribution of TrkB and p75^NTR^ receptors in the adult NSCs response.

## 2 Materials and methods

### 2.1 NSCs cultures

NSCs were obtained from mice with a C57BL6 background. Mice were maintained in a 12-h light/dark cycle with free access to food and water *ad libitum* according to the Animal Care and Ethics Committee of the CSIC. Adult NSCs were isolated from 3-month-old mice after cervical dislocation. The brains were dissected out, and both SVZs from each hemisphere were extracted and cut into small fragments. The pieces were incubated with 0.025% Trypsin-EDTA (Gibco; Cat #25300054) for 30 min at 37°C. The tissue was then transferred to Dulbecco's modified Eagle's medium (DMEM)/F12 (1:1 v/v; Life Technologies, Cat #21331020) and carefully triturated with a fire-polished Pasteur pipette to a single cell suspension. Isolated cells were collected by centrifugation, resuspended in the NSC medium based on DMEM/F12 containing 2 mM Glutamax (Gibco; Cat #35050038), 1X B27 without vitamin A (Gibco; Cat #11500446), 2X antibiotic-antimycotic (Gibco; Cat #15240062), 2 μg/ml heparin (Sigma; Cat #H3393), supplemented with 20 ng/ml epidermal growth factor (EGF; Peprotech, Cat #AF-100-15), and 10 ng/ml fibroblast growth factor (FGF; Peprotech; cat# 100-18B), and maintained in a 95% air−5% CO_2_ humidified atmosphere at 37°C (Bizy and Ferron, 2015; Belenguer et al., 2016). Neurospheres were allowed to develop for 7–10 days in these conditions. Each culture was generated using both SVZs from one adult mouse. Thus, each experimental point in the graphs represents the mean value of the replicates of a single independent animal. For culture expansion, primary neurospheres were disaggregated with Accutase (0.5 mM; Sigma; Cat #A6964) for 10 min at room temperature and washed with the NSC medium without mitogens to generate single-cell suspension. Then, 62.5 cells/μl were plated in the fresh mitogen-completed medium in a 95% air−5% CO_2_ humidified atmosphere at 37°C and maintained for 6–7 passages maximum. In order to determine the self-renewal capacity of the NSCs, secondary neurospheres were disaggregated, NSCs were plated at low density (5 cells/μl) in the fresh mitogen-completed medium, and the number of neurospheres was counted 5 days later. In the self-renewal experiment, four replicates for each culture were used, and the average value was estimated. All these experiments were repeated four times with different cultures. Images of the neurospheres were taken using the PAULA Smart Cell Imager (Leica), and the diameters of the spheres were estimated by ImageJ.

### 2.2 Proliferation and differentiation assays

To estimate proliferation, 62.5 cells/μl were plated after Accutase disaggregation in the fresh mitogen-completed medium in a 95% air−5% CO_2_ humidified atmosphere at 37°C. After 3 days, neurospheres were plated onto cover glasses coated with 1X Matrigel (Corning, Cat #356234) for 15 min, allowing NSC attachment and fixed for staining with 2% paraformaldehyde (PFA) 0.1M phosphate buffer saline pH 7.4 (PBS) for 15 min at 37°C. For the differentiation assay, 80,000 cells/cm^2^ were seeded in Matrigel-coated coverslips and incubated for 2 days (2 DIV) in the NSC culture medium without EGF. The medium was then changed with the fresh medium without FGF and supplemented with 2% fetal bovine serum (FBS; Gibco; Cat #10438-026) for 5 more days (7 DIV) to allow terminal differentiation. Cultures were fixed for staining at 7 days of differentiation with 2% PFA 0.1M PBS for 15 min at 37°C. The BDNF treatment was performed by incubating the NSCs with either 10 ng/ml (low concentration) or 50 ng/ml (high concentration) of Recombinant Human/Murine/Rat BDNF (PeproTech; Cat #450-02) since the single cell suspension is plated. When indicated, NSC cultures were treated with 10 μM ANA-12 (MedChemExpress; Cat #HY-12497) (hereafter referred to as TrkB-i) or 10 μM THX-B (MedChemExpress; Cat #HY-137322) (hereafter referred to as p75-i) at the time of plating to inhibit TrkB or p75^NTR^, respectively. The specificity and selectivity of both antagonists have been previously evaluated (Bai et al., 2010; Cazorla et al., 2011). Control cultures were exposed to 1:1,000 of DMSO (Sigma; Cat.# D5879). In both proliferation and differentiation assays, 10 random images were taken with ~400 cells analyzed for each culture. These experiments were performed four times with independent cultures.

### 2.3 Immunocytochemical procedures

For immunocytochemical staining, fixed cells were permeabilized and blocked with PBS 0.2% Triton X-100 (Sigma; Cat.#X100) containing 10% normal goat serum and 1% glycine (Thermo Scientific; Cat #A13816.36) for 1 h at RT, incubated with primary antibodies, and prepared in the same blocking solution overnight at 4°C. Cells were washed three times with PBS 1X and incubated with secondary antibodies for 1 h at RT. Primary and secondary antibodies and dilutions used are listed in Tables 1, 2, respectively. DAPI (1 μg/ml) was used to counterstain DNA. The samples were washed three times with PBS 1X and mounted with the ImmunoSelect antifading mounting medium (Dianova; Cat #038447). Images were acquired at 20x or 40x magnification with a Leica SP5 confocal microscope. For fluorescence intensity quantification, maximal projection images were generated, and the mean gray intensities of p-TrkB, TrkB, and p75^NTR^ were measured with ImageJ/Fiji software and recorded as arbitrary fluorescence units (a.u.). p-TrkB data were normalized to TrkB intensity.

**Table 1:** List of primary antibodies for immunocytochemistry (ICC) and Western blot (WB).

**Antibody**	**Host**	**Dilution**	**Source**	**Reference**	**RRID**
GFAP	Rabbit	1/500 (ICC)	Abcam	ab7260	AB_305808
Ki67	Rabbit	1/300 (ICC)	Abcam	ab16667	AB_302459
Nestin	Mouse	1/300 (ICC)	Abcam	AB6142	AB_305313
O4	Mouse	1/200 (ICC)	R&D Systems	MAB1326	AB_357617
p75^NTR^	Rabbit	1/200 (ICC); 1/1,000 (WB)	Gift from M. Chao	antiserum #9992	AB_2335792
TrkB	Mouse	1/100 (ICC)	Santa Cruz	sc-136990	AB_2155262
p-TrkB (Y516)	Rabbit	1/200 (ICC)	Invitrogen	PA5-36695	AB_2553666
α-tubulin	Mouse	1/3,000 (WB)	Abcam	ab7291	AB_2241126
βIII-tubulin	Mouse	1/300 (ICC)	Millipore	MAB5564	AB_570921

RRDI, Research Resource Identifiers.

**Table 2:** List of secondary antibodies for immunocytochemistry (ICC) and Western blot (WB).

**Antibody**	**Dilution**	**Source**	**Reference**
Donkey 488 anti-rabbit	1/800 (ICC)	Invitrogen	A21206
Goat 594 anti-mouse	1/800 (ICC)	Invitrogen	A11032
Goat 680RD anti-mouse	1/14,000 (WB)	LI-COR	926-68070
Goat 800CW anti-rabbit	1/14,000 (WB)	LI-COR	925-32211

### 2.4 Western blot

Protein detection by Western blot was performed after protein extraction using 20 mM Tris pH 6.8 (Sigma; Cat.#10708976001) containing 1% Triton X-100 (Sigma), 0.5% sodium dodecyl sulfate (SDS) (Sigma; Cat.#L4509), 1 mM ethylene-dinitrilotetraacetic acid (EDTA) (Merck; Cat.#1.08418.0250), 10 mM β-mercaptoethanol (Sigma; Cat.#M-7154), and 1× cOmplete Mini, EDTA-free protease inhibitor (Roche; Cat.#11836170001). Total protein amount was determined by the Bradford Assay (BioRad; Cat.#500-0006). Proteins were denatured by heat (5 min at 100°C) and loaded in 4–20% precast polyacrylamide gels (BioRad; Cat.#4561095). Proteins were transferred to polyvinylidene difluoride (PVDF) membranes (Merck; Cat.#IPFL00010) and immunoblots were carried out with primary antibodies (Table 1), incubated overnight, and secondary antibodies (Table 2) during 1 h. Antibodies were diluted in Intercept® Blocking Buffer (LI-COR; Cat.#927-60001). After antibodies incubation, the membranes were washed with Tris-buffered saline (TBS) containing 0.1% Tween 20 (Sigma; Cat.#P1379). Finally, protein bands were visualized using the Odyssey CLx Infrared Imaging System (LI-COR).

### 2.5 Gene expression analysis

RNAs were extracted with the RNAeasy mini kit (Qiagen; Cat.# 74104) including DNase treatment, following the manufacturer's guidelines. For quantitative PCR (qPCR), 1 μg of total RNA was reverse transcribed using random primers and SuperScript IV Reverse Transcriptase (ThermoFisher Scientific; Cat# 15317696), following standard procedures. Thermocycling was performed in a final volume of 15 μl, containing 1 μl of cDNA sample (diluted 1:7), and the reverse transcribed RNA was amplified by PCR with appropriate primers from PrimePCR SYBR Green Assay (Cultek; Cat. PB20.11) (see Table 3). qPCR was used to measure gene expression levels normalized to *Rpl27*, the expression of which did not differ between the groups. qPCR reactions were performed in a 7500 real-time PCR equipment (Applied Biosystems). Raw data from this analysis is shown in Supplementary Table 1.

**Table 3:** List of primers for qPCR analysis.

**Gene (protein)**		**Sequence (5′ → 3′)**
*Dcx* (Dcx)	Fw	ACACCCTTGATGGAAAGCAG
	Rv	TGTTCATTGCTTGTGGTCCT
*Nes* (Nestin)	Fw	CTGCAGGCCACTGAAAAGTT
	Rv	GACCCTGCTTCTCCTGCTC
*Ngfr* (p75^NTR^)	Fw	CTAGGGGTGTCCTTTGGAGGT
	Rv	CAGGGTTCACACACGGTCT
*Ntrk2* (TrkB FL)	Fw	CAGTATTAACTCGCTTCTGGC
	Rv	TTCATCCACGTCAAAGGCAG
*Ntrk2.T* (TrkB.T)	Fw	GTCATAGCTAGGTCTAAGTGC
	Rv	GGCAATGGAAAGGGACAAGA
*Olig2* (Olig2)	Fw	CGCAAGCTCTCCAAGATCG
	Rv	CTCACCAGTCGCTTCATCTC
*Rpl27* (Ribosomal protein L27)	Fw	CCCTCCTTTCCTTTCTGCTG
	Rv	GCCATCGTCAATGTTCTTCAC
*S100b* (S100β)	Fw	AAAGTGATGGAGACGCTGGA
	Rv	CTTTGCTGTGCCTCCTCTTG

### 2.6 Statistical analysis

All statistical tests were performed using the GraphPad Prism Software, version 7.00 for Windows. The significance of the differences between groups was evaluated by the two-tailed paired Student *t*-test or one-way ANOVA followed by a Tukey *post-hoc* test. The presence of outlier values was evaluated by Grubb's test. A *p*-value of < 0.05 was considered statistically significant. Data are presented as the mean ± standard error of the mean (SEM) and the number of independent cultures (*n*), and *p*-values are indicated in the figures.

## 3 Results

### 3.1 10 ng/ml BDNF is sufficient to induce self-renew and proliferation of adult NSCs

**Figure 1:**
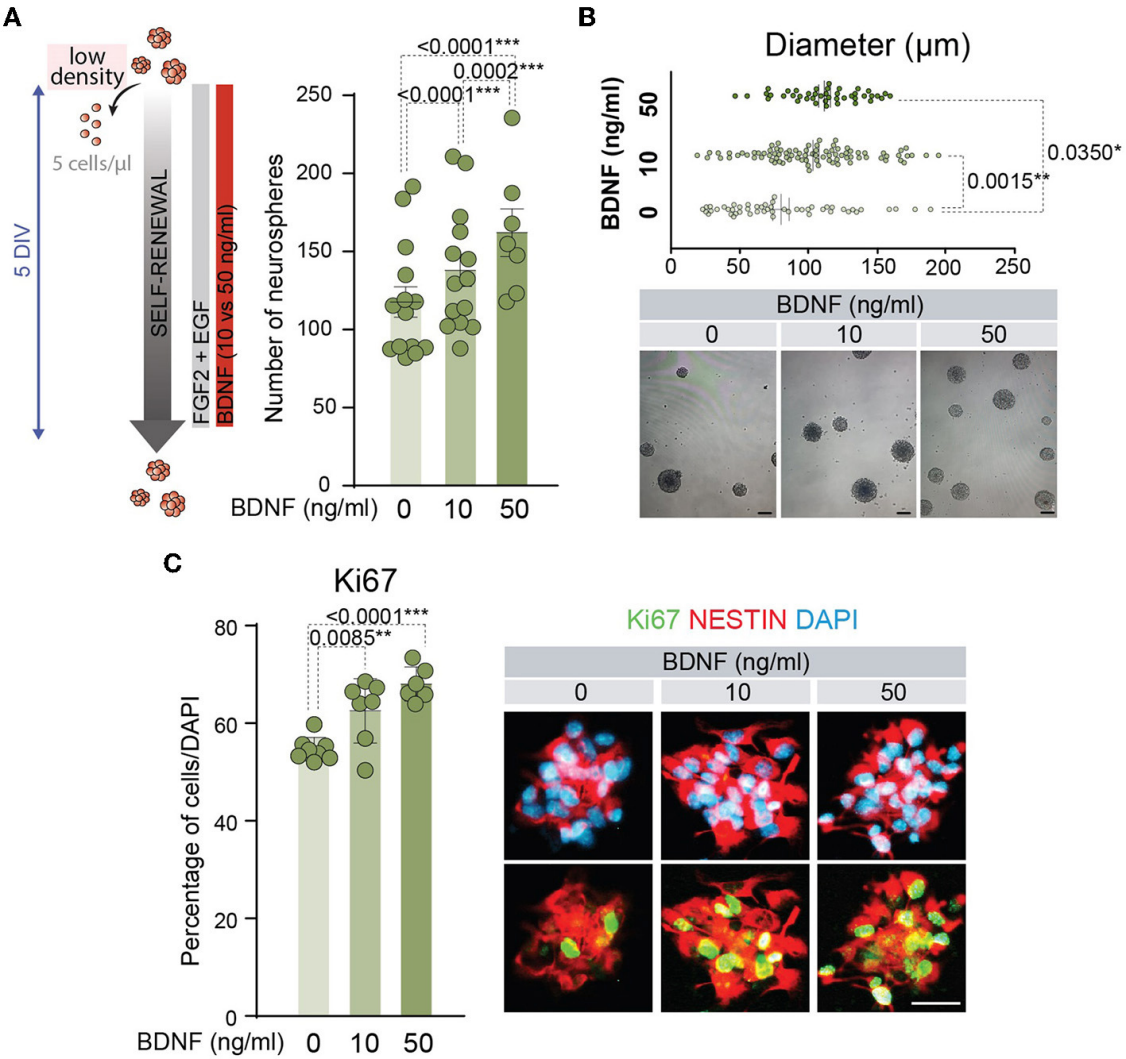
BDNF promotes NSC self-renewal and proliferation. **(A)** Schematic representation of the treatments for different concentrations of BDNF in adult NSCs in the self-renewal assay (left panel). Number of neurospheres after the culture of adult NSCs at low density (5 cells/μl) in the absence or presence of 10 or 50 ng/ml BDNF (right panel). **(B)** Diameter of the neurospheres in the self-renewal assay in the absence or presence of 10 or 50 ng/ml of BDNF (upper panel). Representative images of neurospheres formed in the absence or presence of BDNF treatments (lower panel). **(C)** Percentage of proliferative NSCs at high density (62.5 cells/μl), measured as the proportion of Ki67+ cells, in untreated or BDNF-treated cultures (10 or 50 ng/ml). Immunochemistry images for the proliferative marker Ki67 (green) and the neural precursor marker Nestin (red) in NSCs treated with different concentrations of BDNF are also shown. DAPI was used to counterstain DNA. All error bars show SEM. p-values and the number of samples (circles) are indicated. Only differences that are statistically significant are shown. Scale bars in **(B)** 100 μm; in **(C)** 20 μm.

BDNF has proved to act as a pro-neurogenic factor promoting the proliferation and differentiation of NSCs (Lee et al., 2002; Islam et al., 2009; Chen et al., 2013; Liu et al., 2014; Langhnoja et al., 2021). BDNF activity is mediated by high-affinity binding to the TrkB receptor (Naylor et al., 2002), and this neurotrophic factor is able to interact with low-affinity to p75^NTR^ (Rodriguez-Tebar et al., 1990). Both receptors are expressed in the adult NSCs (Young et al., 2007; Islam et al., 2009; Bath et al., 2012; Faigle and Song, 2013; Vilar and Mira, 2016). A clear positive role for the TrkB pathway has been described in the function of BDNF on the embryonic or P0 NSC proliferation (Islam et al., 2009; Chen et al., 2013), and the proliferative role of p75^NTR^ in the NSCs located in the adult SVZ (Young et al., 2007) remains to be established. To understand the mechanism behind BDNF's effects on the neurogenic population, adult NSCs were treated with two different doses of this neurotrophic factor (10 and 50 ng/ml). We chose these concentrations as the former mainly activates TrkB, while the latter also activates p75^NTR^ since the K_d_ of the interaction of BDNF with p75^NTR^ is approximately 10^−9^ M (~25 ng/ml) (Rodriguez-Tebar et al., 1990). First, self-renewal capacity was tested by determining the number of neurospheres after 5 days of NSCs cultured at low density with low (10 ng/ml) or high (50 ng/ml) concentrations of BDNF (Figure 1A). The presence of BDNF at 10 ng/ml in the NSC cultures significantly increased the number of neurospheres compared to untreated cultures, being this effect potentiated by the addition of BDNF at 50 ng/ml (Figure 1A). This suggests that p75^NTR^ facilitates NSC self-renewal. Moreover, the diameter of these neurospheres was significantly higher in BDNF-treated NSCs (Figure 1B), suggesting an enhancement of NSC proliferation capacity. Both exposures to 10 and 50 ng/ml of BDNF showed a significant increment in the diameter of the neurospheres compared with untreated cultures, whereas no differences were detected between both concentrations of BDNF (Figure 1B). The proliferative capacity of adult NSCs was analyzed by measuring the percentage of positive cells for the cell cycle marker Ki67 (Figure 1C). Both concentrations of BDNF showed a significant increase in the proliferation ratio compared with untreated NSCs. Again, no differences in the percentage of Ki67+ cells were detected between 10 and 50 ng/ml treated cultures (Figure 1C), indicating that the lowest concentration of the neurotrophic factor was sufficient to activate the proliferation pathway.

### 3.2 50 ng/ml BDNF potentiates oligodendrocytic and neuronal differentiation of adult NSCs

**Figure 2:**
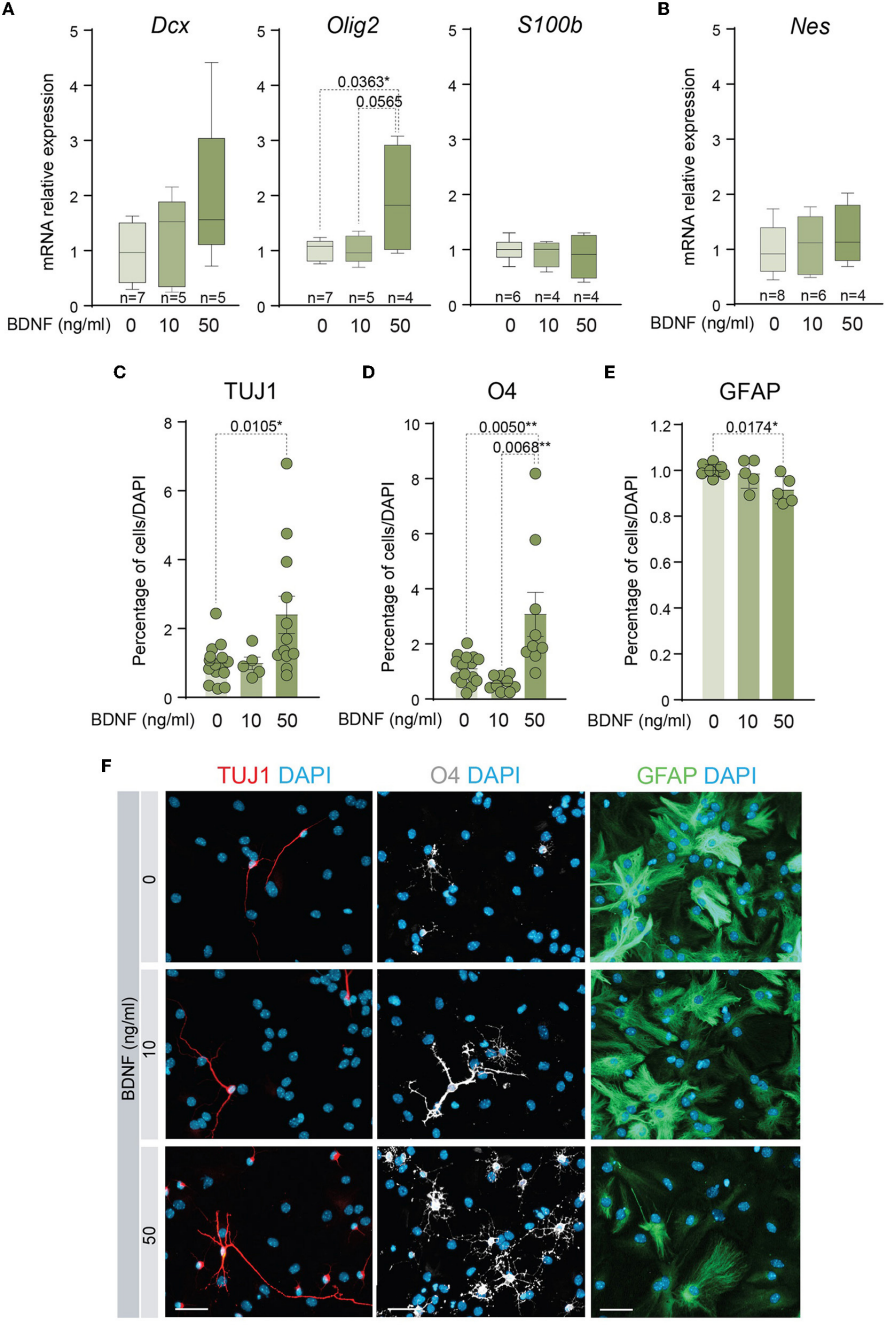
A higher dose of BDNF is required to favor neuronal and oligodendroglial differentiation. **(A)** Boxplots illustrating the expression of the neuronal marker *Dcx*, the oligodendrocytic marker *Olig2*, and the astrocytic marker *S100*β, in BDNF-treated NSCs (0, 10, or 50 ng/ml). *Rpl27* was used as a housekeeping gene. **(B)** Boxplots illustrating the expression of *Nestin* in adult NSCs after being treated with 0, 10, or 50 ng/ml of BDNF. *Rpl27* was used as a housekeeping gene. **(C)** Percentage of neurons, measured as TUJ1+ cells, after 7 days under differentiation-promoting condition in the absence or presence of 10 or 50 ng/ml of BDNF. **(D)** Percentage of oligodendrocytes, measured as O4+ cells, after 7 days under differentiation-promoting condition in the absence or presence of 10 or 50 ng/ml of BDNF. **(E)** Percentage of astrocytes, measured as GFAP+ cells, after 7 days under differentiation-promoting condition in the absence or presence of 10 or 50 ng/ml of BDNF. **(F)** Immunocytochemistry images for TUJ1 (red), O4 (gray), or GFAP (green) in NSCs after 7 DIV of differentiation in the absence or presence of 10 or 50 ng/ml of BDNF. DAPI was used to counterstain DNA. In **(C–E)**, *p*-values and the number of samples (circles) are indicated, and all error bars show SEM. Only differences that are statistically significant are shown. Scale bars in **(F)** 30 μm.

Several studies have shown that BDNF exerts a positive effect on the differentiation of NSCs into neurons (Chen et al., 2013; Liu et al., 2014) and oligodendrocytes (Chen et al., 2013; Langhnoja et al., 2021). In accordance, the mRNA levels of relevant differentiation markers were analyzed by qPCR in cDNAs obtained from adult NSCs. This analysis indicated that the expression of the neuronal marker *Dcx* showed a tendency to increase and the oligodendrocyte marker *Olig2* was significantly upregulated in the NSCs treated with 50 ng/ml of BDNF, suggesting that treatment with a high dose of BDNF predisposes NSCs toward a more committed state. Instead, the presence of 10 ng/ml of BDNF in the medium was not sufficient to increase the levels of mRNA of these lineage genes (Figure 2A). Neither the expression of the mRNA encoding the astrocytic marker *S100*β (*S100b*) nor the neural precursor gene *Nestin* (*Nes*) showed differences between untreated and BDNF-treated NSCs (Figures 2A, B). To test if the upregulation of the neuronal and oligodendrocytic genes in the adult NSCs after 50 ng/ml BDNF treatment drove an increment in the percentage of neurons and oligodendrocytes in differentiating conditions, the number of TUJ1+, O4+, and GFAP+ cells, representing neurons, oligodendrocytes, and astrocytes, respectively, were estimated after seven DIV in NSCs maintained in differentiation conditions. The percentage of neurons and oligodendrocytes were increased in the 50 ng/ml BDNF treated cultures, at the expense of astrocyte generation, which decreased in this condition compared with untreated cells (Figures 2C–F). Moreover, the treatment with the low dose of BDNF (10 ng/ml) did not alter the differentiation capacity of adult NSCs regarding untreated cultures (Figures 2C–F), thus requiring a higher concentration of BDNF to activate the differentiation pathway. These data, together with those from the proliferation analysis shown above, suggest different mechanisms for BDNF to promote proliferation or differentiation in a dose-dependent manner.

### 3.3 BDNF promotes the expression of TrkB- and p75^*NTR*^-specific mRNAs, the phosphorylation of TrkB, and the upregulation of p75^*NTR*^

**Figure 3:**
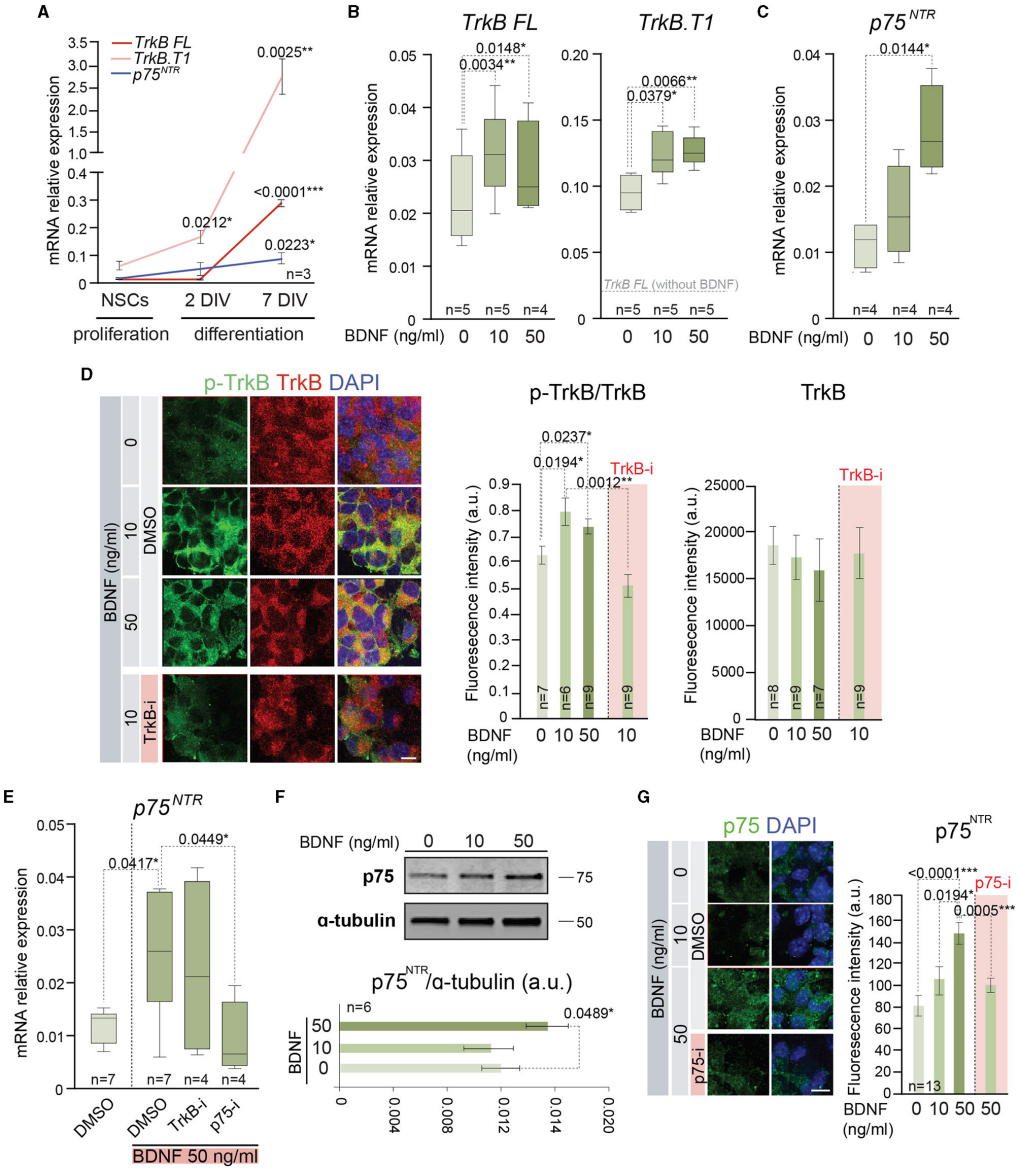
BDNF induces the expression of functional TrkB and p75^NTR^ receptors. **(A)** Expression of mRNA encoding the full-length (*TrkB FL*) and the truncated (*TrkB.T1*) isoform of *Ntrk2* gen and p75^NTR^ (*Ngfr*) in adult NSCs maintained in the absence of exogenous BDNF in proliferation-promoting conditions and during the differentiation process [after 2 days *in vitro* (DIV) and 7 DIV]. **(B)** Boxplots illustrating the expression of *TrkB FL* and *TrkB.T1* isoform of *Ntrk2* gen in adult NSCs after being treated with 0, 10, or 50 ng/ml of BDNF. **(C)** Boxplots illustrating the expression of *Ngfr* (*p75*^*NTR*^) in BDNF-treated NSCs (0, 10, or 50 ng/ml). **(D)** Representative high-magnification images illustrating the immunostaining for p-TrkB in Y516 (green) and TrkB (red) in untreated or BDNF-treated (10 or 50 ng/ml) neurospheres as well as 10 ng/ml BDNF-treated neurospheres with the TrkB antagonist (TrkB-i). Vehicle: DMSO (left panel). Quantification of p-TrkB/TrkB and TrkB fluorescence intensity (in arbitrary units, a.u.) in these cultures (middle and right panels). **(E)** Boxplots illustrating the expression of p75^NTR^ receptor, *Ngfr*, in adult NSCs in the absence or presence of 50 ng/ml of BDNF and treated with the antagonists TrkB-i or p75-i. DMSO was used as a control. **(F)** Immunoblot for p75^NTR^ protein in NSC cultures treated with 0, 10, or 50 ng/ml of BDNF (upper panel). Quantification in the Western blot of p75^NTR^ relative to α-tubulin protein (bottom panel). **(G)** Representative images illustrating the immunostaining for p75^NTR^ in untreated or treated NSCs with 10 or 50 ng/ml of BDNF as well as 50 ng/ml BDNF-treated cells with the p75^NTR^ antagonist (p75-i). Quantification of p75^NTR^ fluorescence intensity in arbitrary units is shown as mean ± SEM (*n* = 13). In **(A–C, E)**, *Rpl27* was used as a housekeeping gene. DAPI was used to counterstain DNA in **(D, G)**. In **(A, D, F)**, error bars show SEM. In all panels, *p*-values and the number of samples are indicated. Only differences that are statistically significant are shown. Scale bars: 20 μm **(D)**; 10 μm **(G)**.

The previous results of proliferation and differentiation of the adult NSCs in the presence of low or high doses of BDNF could be explained by the use of different signaling mechanisms to activate each cellular process. Precisely, BDNF presents high-affinity binding to TrkB (Naylor et al., 2002) and low-affinity binding to p75^NTR^ (Rodriguez-Tebar et al., 1990), two receptors that are expressed by NSCs, showing a dynamic pattern of expression during proliferation and differentiation of these cells (Figure 3A). To understand if the different cellular response of BDNF in a dose-dependent manner could be due to the intervention of different receptors/pathways, adult NSCs were treated with 10 or 50 ng/ml of BDNF, and the gene expression of both receptors, *Ntrk2* (TrkB) and *Ngfr* (p75^NTR^), was measured by qPCR (Figures 3B, C). To this aim, BDNF was added after neurosphere disaggregation, and the expression of these receptors was analyzed in the newly formed neurospheres after 5 days in the presence of the neurotrophin. The *Ntrk2* gene encodes three receptor isoforms generated by alternative splicing, the full-length isoform (TrkB FL), and two truncated versions of the protein lacking the kinase domain, with TrkB.T1 being the most expressed in the NSCs from the SVZ (Islam et al., 2009; Vilar and Mira, 2016). Thus, the expression of the transcripts encoding both TrkB FL and TrkB.T1 (*TrkB FL* and *TrkB.T1*, respectively) was analyzed in adult NSCs grown in the absence or presence of 10 or 50 ng/ml BDNF (Figure 3B). The presence of BDNF in the culture medium resulted in a significant increment of both *TrkB FL* and *TrkB.T1* expressions, regardless of the BDNF concentration (Figure 3B), suggesting that its expression is regulated by the activation of TrkB. As previously shown (Islam et al., 2009), the expression of *TrkB.T1* was higher than that of *TrkB FL* (Figure 3B). In contrast to its mRNA levels, the expression of the TrkB protein using an antibody recognizing the extracellular domain (i.e., recognizing all TrkB isoforms) was not observed to show an increased response to BDNF (Figure 3D), suggesting that post-transcriptional mechanisms regulate TrkB protein expression. As expected, exposure of neurospheres to BDNF resulted in the increase of TrkB phosphorylation in Y516 (Figure 3D), a residue that becomes phosphorylated upon TrkB activation (Mazzaro et al., 2016). This activation of TrkB signaling in NSCs confirms previous published data suggesting TrkB activation in NSCs (Chen et al., 2017). Moreover, the application of the selective TrkB antagonist ANA-12 (TrkB-i) (Cazorla et al., 2011) to neurospheres treated with 10 ng/ml BDNF resulted in the reduction of Y516 TrkB phosphorylation to basal levels (Figure 3D). In contrast to *TrkB FL* and *TrkB.T1* expressions, the expression of *Ngfr* was significantly upregulated in the NSC cultures only after high-dose exposure to BDNF (Figure 3C), indicating that the presence of high levels of BDNF promotes the activation of a signaling pathway resulting in the expression of *Ngfr*. The requirement for the dose of BDNF suggests that the upregulation of p75^NTR^ is modulated by its own activation. To confirm this hypothesis, the expression of *Ngfr* was measured in NSCs treated with 50 ng/ml BDNF in the presence of TrkB-i or the selective p75^NTR^ antagonist THX-B (Bai et al., 2010) (p75-i) (Figure 3E). The presence of TrkB-i did not change the *Ngfr* mRNA levels when NSCs were treated with 50 ng/ml of BDNF, and the expression of *Ngfr* was not upregulated after 50 ng/ml BDNF treatment in the presence of p75-i (Figure 3E), showing that the increment in the expression of the p75^NTR^ receptor was regulated by the interaction of BDNF with this receptor. The increment in *Ngfr* mRNA at 50 ng/ml of BDNF treatment was confirmed at the protein level by Western blot (Figure 3F) and immunocytochemistry (Figure 3G), using a previously characterized antibody (Huber and Chao, 1995).

### 3.4 TrkB and p75^*NTR*^ are required for BDNF-mediated self-renewal and proliferation of adult NSCs

**Figure 4:**
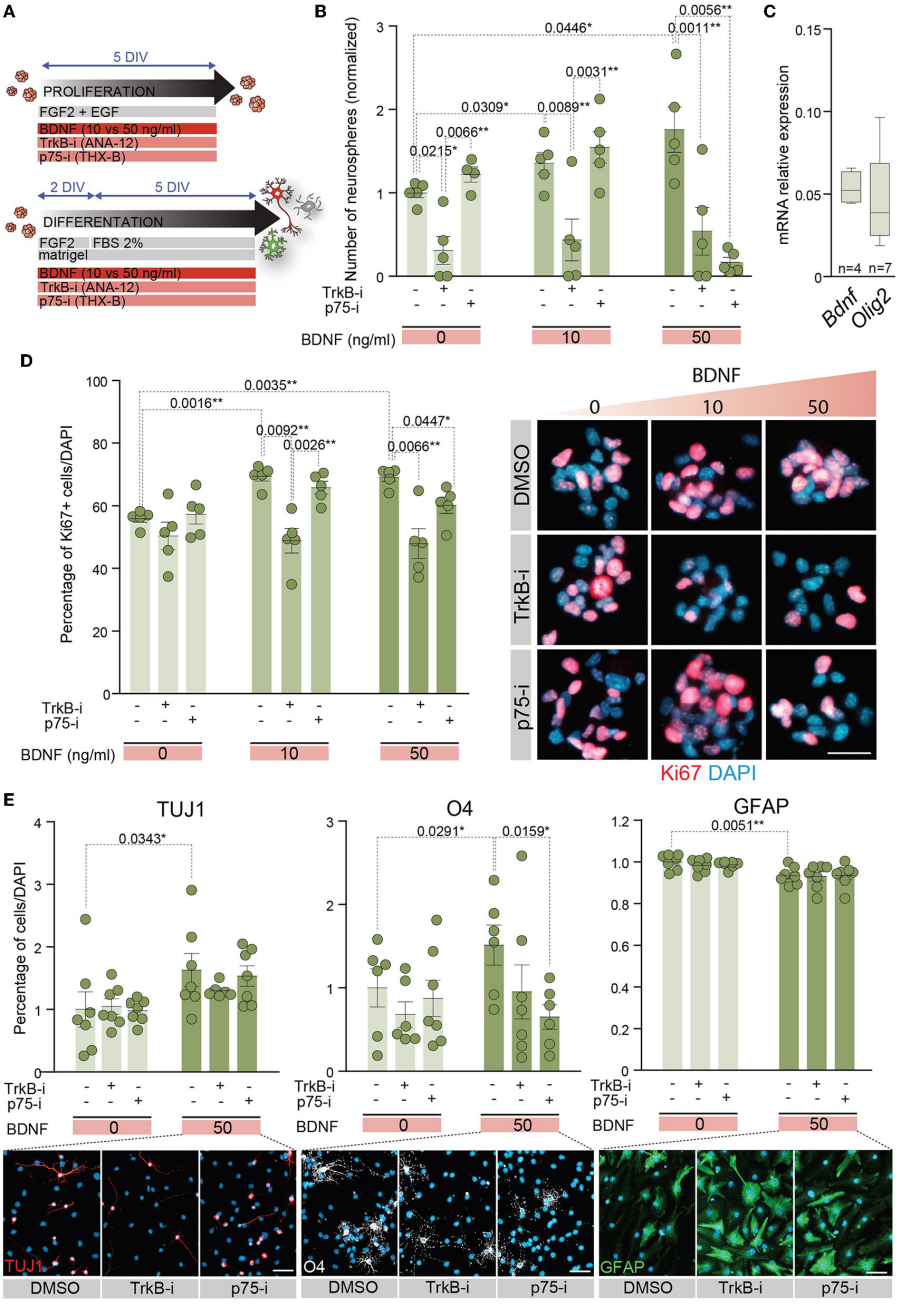
p75^NTR^ regulates adult NSC proliferation and differentiation in a higher dose of BDNF context. **(A)** Schematic representation of the treatments for TrkB and p75^NTR^ inhibition in adult NSCs in proliferation or differentiation conditions. **(B)** Number of neurospheres in 0, 10, or 50 ng/ml BDNF treatments in the presence of the TrkB antagonist, TrkB-i, or the p75 antagonist, p75-i. As a control, DMSO was added to the cultures without antagonists. **(C)** Boxplots illustrating the expression of *Bdnf* and *Olig2* by qPCR in untreated adult NSCs. *Olig2* expression is shown as a positive control of a neural expressed gene. *Rpl27* was used as a housekeeping gene. **(D)** Percentage of Ki67 positive cells in NSCs treated with 0, 10, or 50 ng/ml of BDNF in the presence of the antagonists TrkB-i or p75-i (left panel). Immunocytochemistry images for Ki67 (red) in these conditions (right panel). **(E)** Percentage of TUJ1+ neurons, O4+ oligodendrocytes, and GFAP+ astrocytes after 7 DIV under differentiation-promoting conditions in 50 ng/ml BDNF-treated or untreated NSCs in the presence of TrkB-i or p75-i antagonists (upper panels). As a control, DMSO was added to the cultures without antagonists. Immunocytochemistry images for the lineage markers TUJ1 (red), O4 (gray), and GFAP (green) in these conditions are also shown (lower panels). DAPI was used to counterstain DNA. Error bars show SEM. *p*-values and the number of samples (circles) are indicated. Only differences that are statistically significant are shown. Scale bars in **(D)** 20 μm; in **(E)** 30 μm.

The expression data of TrkB and p75^NTR^ in proliferating and differentiating conditions suggest that both receptors are involved in NSC behavior. To determine the implications of TrkB and p75^NTR^ in these processes, NSCs were treated with TrkB-i and p75-i, respectively (Figure 4A). NSCs were cultured at low density to evaluate self-renewal capacity in the absence or presence of 10 or 50 ng/ml of BDNF as above, using 10 μM of TrkB-i or 10 μM of p75-i to inhibit TrkB or p75^NTR^ specifically (Figure 4B). Control NSCs were treated with DMSO. The presence of TrkB-i in the medium revealed that the TrkB pathway is essential for NSCs to self-renew, independently of the presence of exogenous BDNF, a finding consistent with the expression of *Bdnf* by the adult NSCs (Figure 4C). Blocking this receptor significantly decreased the number of neurospheres in 0, 10, and 50 ng/ml of BDNF treatments (Figure 4B). These data were consistent with previous results showing a decrease of newly born neurons in the OB of TrkB heterozygous mice (Bath et al., 2008). In contrast, treatment of NSCs with p75-i in the absence of BDNF showed no effect on the self-renewal capacity of the NSCs (Figure 4B). The presence of 10 ng/ml of BDNF jointly with this antagonist did not alter this ability either (Figure 4B) indicating that lower concentrations of BDNF act through the TrkB pathway. However, treatment with 50 ng/ml of BDNF in the presence of the p75^NTR^ antagonist resulted in a decrease in the number of neurospheres (Figure 4B), indicating that the higher concentration of BDNF activated a TrkB/p75^NTR^-dependent pathway that becomes necessary to control NSC self-renewal. Previous studies demonstrated that TrkA formed complexes with p75^NTR^, increasing the affinity and selectivity of NGF binding (Hempstead et al., 1991). Another study showed that BDNF induces TrkB association with p75^NTR^ in embryonic hippocampal neurons after TrkB activation (Zanin et al., 2019). Importantly, this latter study demonstrated that p75^NTR^ is necessary for optimal TrkB signaling and function through the PI3K pathway in embryonic neurons (Zanin et al., 2019). In contrast to these studies, where p75^NTR^ optimizes the signaling capacity of the Trk family receptors, our observation suggests that a novel functional interaction between p75^NTR^ and TrkB exists in the adult NSCs as the blockade of p75^NTR^ prevents TrkB function.

The proliferation capacity of adult NSCs was also analyzed in the presence of the receptor antagonists (Figure 4D). NSCs were plated in proliferation-promoting conditions and treated with different doses of BDNF. The percentage of proliferating cells was determined by the number of Ki67+ cells. The treatment with either antagonist in the absence of exogenous BDNF showed no alterations in the percentage of proliferative NSCs (Figure 4D). The presence of the TrkB-i in NSCs treated with low or high concentrations of BDNF prevented the increase in the percentage of Ki67+ cells induced by this neurotrophin, reaching the untreated culture levels (Figure 4D). However, the presence of the p75-i decreased the Ki67 percentage to untreated culture levels only in NSCs treated with 50 ng/ml BDNF (Figure 4D), demonstrating activation of the p75^NTR^ pathway when BDNF levels are high, leading to increased proliferation.

### 3.5 p75^*NTR*^ is required for BDNF-mediated differentiation of adult NSCs into oligodendrocytes

Since BDNF-mediated differentiation requires high levels of BDNF (Figures 2C–F), we investigated whether the p75^NTR^ activation observed under proliferative conditions was also required to achieve terminal differentiation of adult NSCs. Thus, NSCs were differentiated in the presence of a high concentration of BDNF and either of the antagonists TrkB-i and p75-i (Figure 4E). In the absence of BDNF, no alterations were detected in the percentage of neurons, oligodendrocytes, and astrocytes after the receptor blockage (Figure 4E). The differentiation of NSCs with 50 ng/ml of BDNF increased the percentage of neurons and oligodendrocytes at the expense of astrocytes, as previously demonstrated. However, only p75^NTR^ inhibition with p75-i was able to rescue the proportion of oligodendrocytes observed in the control cultures with statistical significance (Figure 4E). No statistically significant alterations were observed in the percentage of astrocytes and neurons with the TrkB-i and p75-i antagonists. However, a decrease in the proportion of oligodendrocytes with TrkB-i antagonist in 50 ng/ml BDNF-treated cultures was detected, not reaching statistical significance (Figure 4E).

## 4 Discussion

**Figure 5:**
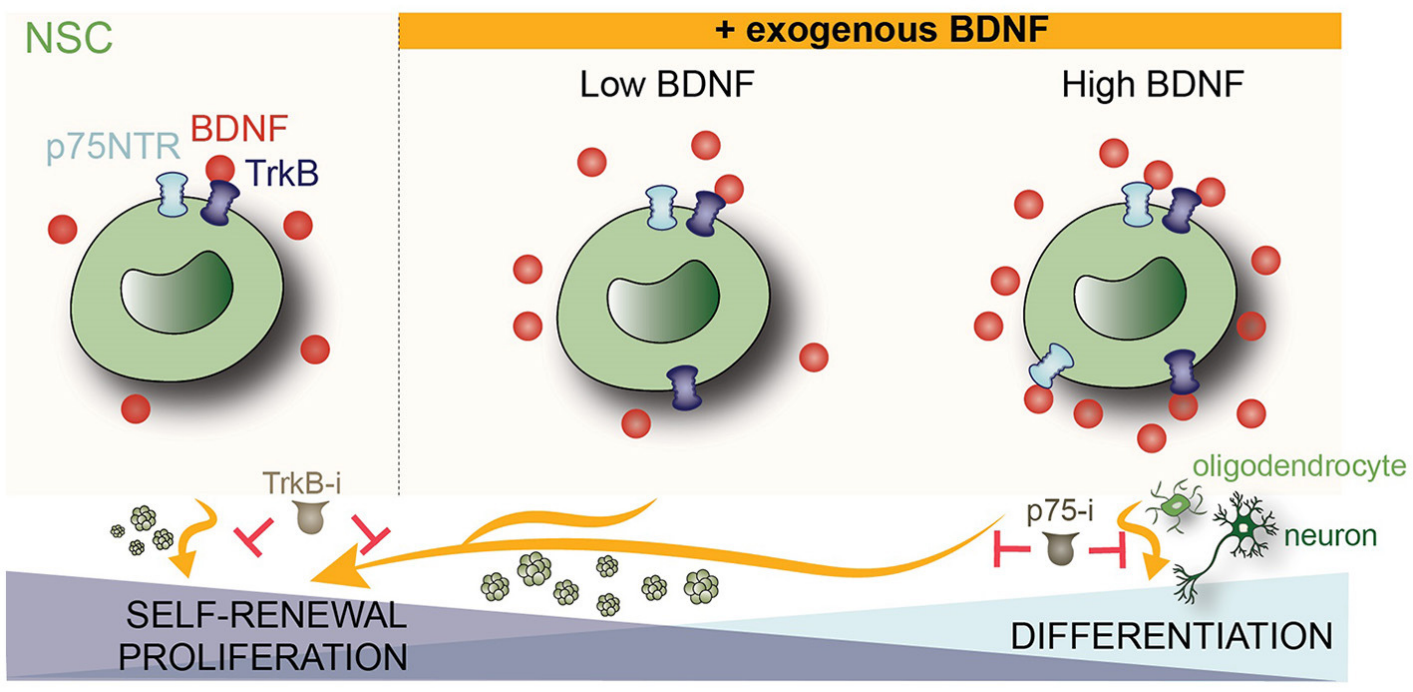
Scheme showing how BDNF regulates self-renewal, proliferation, and differentiation of adult NSCs by TrkB and p75^NTR^ receptors. In homeostasis conditions **(left)**, NSCs require TrkB signaling to maintain their self-renewal capacity. Inhibition of this receptor by a specific TrkB antagonist (TrkB-i) reduces the formation of neurospheres. Exposure to low concentrations of BDNF (10 ng/ml) increases the self-renewal and proliferative activity of the adult NSCs, which is prevented by the TrkB antagonist. Exposure to high concentrations of BDNF (50 ng/ml) **(right)** induces the upregulation of p75^NTR^, thus becoming self-renewal and proliferation dependent on p75^NTR^. The high concentration of BDNF also promotes NSC commitment to neuronal and oligodendroglial lineage. This increment of oligodendrocytes is prevented by a p75^NTR^-specific antagonist (p75-i). Thus, in an exogenous BDNF context, TrkB regulates self-renewal and proliferation in NSCs isolated from the adult SVZ, whereas p75^NTR^ is implicated in self-renewal, proliferation, and differentiation in the presence of a high dose of the neurotrophin.

We have shown in this study that BDNF facilitates self-renewal and cell cycle progression in NSCs isolated from the SVZ of adult mice. These processes are mediated by the TrkB/TrkB.T1 receptors as they can be blocked by ANA-12 (TrkB-i), an inhibitor that interacts with the binding domain of BDNF in the extracellular domain of these receptors (Cazorla et al., 2011). Interestingly, both self-renewal and cell cycle progression become dependent on p75^NTR^ when the concentration of BDNF is high enough to activate this latter receptor. Under this condition, BDNF does not exert proliferative effects if the p75^NTR^ function is pharmacologically blocked. In addition, we have demonstrated that BDNF induces the differentiation of NSCs into oligodendrocytes through a p75^NTR^-dependent mechanism as it requires a BDNF concentration above its K_d_ for the binding to p75^NTR^ and can be pharmacologically blocked with a p75^NTR^-specific inhibitor. We have also shown that BDNF triggers neuronal differentiation when applied at a high dose (Figure 5).

Our results indicate that BDNF is required for NSC self-renewal. This effect is dose-dependent since a significantly higher number of neurospheres can be observed in the presence of 50 ng/ml BDNF when compared to 10 ng/ml. This facilitation has been previously described for the BDNF-dependent survival of rat hippocampal neurons (Zanin et al., 2019). The mechanism by which 50 ng/ml BDNF potentiates NSC self-renewal might depend on the observed upregulation of p75^NTR^ expression at this high BDNF concentration. This increase in p75^NTR^ at high BDNF dose is reminiscent of the effect of NGF in astrocytes, which can also upregulate p75^NTR^ expression in these cells (Kumar et al., 1993). In the adult SVZ, the p75^NTR^-positive population contains all of the neurosphere-producing precursor cells (Young et al., 2007). Therefore, we suggest that the observed increase of p75^NTR^ in our cultures when treated with 50 ng/ml BDNF is likely due to the activation of p75^NTR^ and the upregulation of its levels in all the neurosphere-constituting cells. In fact, the upregulation of the mRNA and protein p75^NTR^ levels was blocked in the presence of p75-i, indicating activation of this receptor after high-dose BDNF treatment to promote its own expression.

In this study, we have demonstrated that BDNF facilitates the proliferation of adult mouse NSCs *in vitro*. This observation is consistent with the finding that BDNF stimulates the proliferation of newborn NSCs (Chen et al., 2013), human iPSCs-derived NPCs (Pansri et al., 2021), and embryonic neural precursors (Bartkowska et al., 2007). The stimulation of proliferation triggered by BDNF likely depends on TrkB since the use of TrkB-i prevents it. This is consistent with the known activation by TrkB of the Ras–Raf–MEK–ERK signaling pathway (Reichardt, 2006), which favors cell cycle progression when ERK translocates to the nucleus and phosphorylate transcription factor substrates that are responsible for the mitogenic response (Mebratu and Tesfaigzi, 2009). This is consistent with our observation that BDNF induces the phosphorylation of TrkB in Y516, a residue known to participate in the latter signaling pathway (Fan et al., 2020). Nevertheless, TrkB.T1 may also participate in the facilitation of NSC proliferation by BDNF as the truncated form of TrkB has been suggested to induce BDNF-dependent proliferative effects on both embryonic NSCs (Islam et al., 2009) and embryonic neural progenitors (Tervonen et al., 2006).

Our results indicate that the intrinsic ability of TrkB to confer both self-renewal and proliferative capacity to NSCs (i.e., the proliferative capacity that would be observed upon pharmacological inhibition of p75^NTR^) becomes unexpectedly abolished when BDNF is added at 50 ng/ml. We explain this result in terms of the differential capacity of BDNF to activate p75^NTR^ depending on its concentration (Rodriguez-Tebar et al., 1990). We propose that the activation of p75^NTR^ with 50 ng/ml BDNF seems to permanently modify the proliferative signaling of TrkB. We refer to this effect as “co-receptor dependence for TrkB signaling.” The mechanism of acquisition of this novel co-receptor dependence is currently unknown. However, it should not derive from a different mode of TrkB activation by the higher BDNF concentration since the binding capacity of BDNF to the high-affinity TrkB receptor has already reached a plateau at the range of 10–50 ng/ml (Rodriguez-Tebar et al., 1990). This co-receptor dependence for TrkB signaling might be physiologically relevant *in vivo*, in neurogenic regions where local enrichment of BDNF results in the upregulation of p75^NTR^ and the modulation of TrkB/p75^NTR^ signaling. Our results are consistent with previous studies in postnatal hippocampal NSCs demonstrating the implication of p75^NTR^ in the proliferation capacity of these cells since the p75^NTR^-ligand proNGF inhibits proliferation of the NCSs (Guo et al., 2013). As proNGF cannot interact with TrkB, it likely prevents the functional interaction of p75^NTR^ with the latter in response to endogenously produced BDNF. This effect was also abolished in p75^NTR^ knock-out mice (Guo et al., 2013), thus providing genetic evidence that this receptor is involved in the proliferation of NSCs.

Our results also indicate that the pharmacological inhibition of TrkB results in a dramatic reduction in the number of neurospheres even in the absence of added BDNF, suggesting that low levels of this neurotrophin may be released by the NSCs facilitating their self-renewal. Indeed, previous studies have shown BDNF expression in the SVZ (Galvão et al., 2008) and embryonic NSCs (Blurton-Jones et al., 2009). We have shown that *Bdnf*-specific mRNA is transcribed by adult NSCs, a finding consistent with a previous study by Goldberg et al. (2015). In contrast, TrkB inhibition in the Ki67 proliferation assay without exogenous BDNF does not lead to a significant reduction in cell cycle progression. The main difference between both results is the density of the NSCs that were used. In the proliferative assay, high NSC density was employed, while in the self-renewal assay, NSCs were plated at low density. Therefore, one explanation for this discrepancy may derive from a hypothetical capacity of TrkB to stimulate either the expression or function of the cell adhesion molecules involved in the generation of the neurospheres (Zhou et al., 1997). Consequently, NSCs would not adhere to each other to generate multicellular structures in the presence of TrkB-i.

In this study, we have demonstrated that BDNF induces the differentiation of adult NSCs *in vitro* into oligodendrocytes and neurons, as previously shown to take place in newborn NSCs (Chen et al., 2013; Langhnoja et al., 2021). This is consistent with the capacity of BDNF to promote the progression of oligodendrocyte lineage and to enhance myelination through the p75^NTR^ receptor (Cosgaya et al., 2002). The studies by Chen et al. (2013) and Langhnoja et al. (2021) mentioned above did not compare the roles of TrkB and p75^NTR^ in this process. Nevertheless, we note that high concentrations of BDNF were used by these authors to detect a potent differentiative effect on newborn NSCs (25 and 50 ng/ml BDNF, respectively). We therefore decided to explore which BDNF receptor is responsible for the differentiative effect of BDNF. Our results indicate that BDNF induces differentiation through p75^NTR^-dependent signaling based on two lines of evidence. On the one hand, this effect could not be observed with 10 ng/ml BDNF, a concentration that is insufficient to activate p75^NTR^ (Rodriguez-Tebar et al., 1990). On the other hand, the use of p75-i, in contrast to TrkB-i, significantly blocked BDNF-dependent oligodendrocyte differentiation. These results agree with the known inhibition of oligodendrogenesis in a p75^NTR^-dependent manner since this process was blocked in the presence of proNGF and p75^NTR^ knock-out mice (Guo et al., 2013).

We have observed that the p75^NTR^-specific inhibitor was not able to prevent neuronal differentiation *in vitro*, which is consistent with the observation that p75^NTR^ null mice had nearly identical levels of surviving BrdU-positive cells in the OB relative to wild-type mice 28 days after DNA labeling with this nucleotide analog (Bath et al., 2008). This contrasts with our observation that 50 ng/ml BDNF, but not 10 ng/ml BDNF, is required to induce neuronal differentiation in our cultures. In this regard, we note that a great statistical error can be observed in the increase of TUJ1-positive cells when the NSCs are treated with 50 ng/ml BDNF under differentiative conditions (Figures 2C, 4E). Therefore, it cannot be strongly concluded that BDNF triggers a clear effect on neuronal differentiation through p75^NTR^.

Our results are consistent with the observation that intraventricular administration of BDNF increases the number of newly generated neurons in the adult rat olfactory bulb (Zigova et al., 1998; Benraiss et al., 2001; Henry et al., 2007). They are also consistent with the reduction in the number of newborn neurons that is observed in the OB of mice lacking one copy of the Bdnf gene (Bath et al., 2008). They are also consistent with the claim that TrkB is not essential for adult SVZ neurogenesis (Galvão et al., 2008). Mechanistically, the observation that neurotrophin binding to p75^NTR^ modulates Rho activity and axonal outgrowth (Yamashita et al., 1999) and that developmental biology is one of the enriched pathways associated with p75^NTR^ function (Sajanti et al., 2020) may explain the differentiative effect of BDNF-dependent activation of p75^NTR^ in adult NSCs.

Taken together, our results provide the basis to understand the role of BDNF in the homeostasis of SVZ-derived adult NSCs and the implications of this relevant neurotrophin in pathological conditions as we have clarified the differential contribution of TrkB and p75^NTR^ to BDNF-dependent self-renewal, proliferation, and differentiation of adult NSCs. Furthermore, our results reveal an undescribed mechanism based on a co-receptor dependence for TrkB signaling in the regulation of self-renewal and proliferation of adult NSCs that may be a clue to understand BDNF effects in the neurogenic niche.

## Data availability statement

The original contributions presented in the study are included in the article/Supplementary material, further inquiries can be directed to the corresponding author/s.

## Ethics statement

The animal study was approved by Comité de Ética (Consejo Superior de Investigaciones Científicas). The study was conducted in accordance with the local legislation and institutional requirements.

## Author contributions

AL-U: Conceptualization, Formal analysis, Methodology, Writing–original draft, Writing–review & editing. JF: Conceptualization, Funding acquisition, Supervision, Writing–review & editing.
